# Early-Life Exposure to Bisphenol A Induces Liver Injury in Rats Involvement of Mitochondria-Mediated Apoptosis

**DOI:** 10.1371/journal.pone.0090443

**Published:** 2014-02-28

**Authors:** Wei Xia, Ying Jiang, Yuanyuan Li, Yanjian Wan, Juan Liu, Yue Ma, Zhenxing Mao, Huailong Chang, Gengqi Li, Bing Xu, Xi Chen, Shunqing Xu

**Affiliations:** Key Laboratory of Environment and Health, Ministry of Education and Ministry of Environmental Protection, and State Key Laboratory of Environmental Health (Incubating), School of Public Health, Tongji Medical College, Huazhong University of Science and Technology, Wuhan, China; Universidad Miguel Hernández de Elche, Spain

## Abstract

Exposure to bisphenol A (BPA), a monomer widely used to manufacture polycarbonate plastics, has been reported to be associated with abnormalities of liver function and hepatic damage. However, the molecular mechanism under the pathogenesis of hepatic injury is unclear. In this study, the effect of perinatal exposure to BPA at the reference dose of 50 µg/kg/day on the apoptotic index in the liver of rat offspring was investigated. Increased levels of ALT and enhanced cell apoptosis were observed in the liver of rat offspring at 15 and 21 weeks, and significantly increased activity of caspase-3 and caspase-9 and elevated levels of cytochrome c were also confirmed. In addition, significant change in the expression levels of Bcl-2 and Bax were found in BPA-treated offspring at 21 weeks. For *in vitro* experiments, liver mitochondria were isolated from neonatal rats and were treated with BPA. BPA treatment led to a significant increase in mitochondrial permeability transition. Moreover, the supernatant from BPA-treated mitochondria significantly increased apoptotic changes in nuclei isolated from liver tissue. In conclusion, the study demonstrates that BPA induces mitochondria-mediated apoptosis in hepatic cells, which may contribute to long-term hepatotoxicity induced by early-life exposure to BPA.

## Introduction

Bisphenol A (BPA) is an environmental chemical that has been widely used in the manufacture of polycarbonate plastics and epoxy resins for many years. Due to its major applications in the production of plastic food or beverage containers and the coating of food cans, people of different ages are inevitably exposed to BPA in daily life. BPA has been detected in human placenta [1], cord blood [2], amitotic fluid [3], [4], fetal liver [5] and breast milk [6], making exposure of human neonates and infants a very real concern. A number of studies have revealed that maternal exposure to BPA during gestation and/or lactation induced adverse effects in offspring, such as affecting the development of reproductive organ [7], [8] and metabolic function [9]–[12].

In humans and animals, the liver is the primary organ responsible for BPA metabolism to its glucurono-conjugated form [13]. From epidemiological studies, higher urinary concentration of BPA is associated with serum markers of abnormal liver function in human adults [14]. *In vivo* animal data suggest that BPA is able to induce reactive oxygen species generation and disturb enzyme activity in liver of rats [15], [16], and induce some degree of fat infiltration in mice liver [17], [18]. *In vitro*, BPA is known to induce cytotoxic effect on cultured rat hepatocytes at high doses [19], [20]. However, whether perinatal exposure to BPA at environmentally relevant doses may exert these effects on liver and the pathogenesis of hepatoxicity induced by BPA remains to be determined.

Although there has been a focus on BPA as an endocrine disruptor due to its estrogenic activity, there might be other mechanisms that explain the effects of BPA. Mitochondrial dysfunction induced by BPA, including decrease in mitochondrial transmembrane potential and altered cellular oxidation-reduction, have been reported in the isolated rat hepatocytes [19], and in human HepG2 cell [21], suggesting mitochondria are a target of BPA at organelle level. It’s known that mitochondria play a crucial role in apoptosis by releasing the intermembrane space proteins, such as cytochrome c (Cyt c), which is a key mediator of apoptosis for activation of caspase in the cytosol [22], [23]. Additionally, an alteration in the ratio of pro-apoptotic to anti-apoptotic proteins of the Bcl-2 family, resided on the outer membrane of mitochondria, may modulate the release of apoptogenic proteins [24]. Recently, BPA-induced apoptosis has been demonstrated in cultured liver cells [19], [25]. However, the mechanism of action of BPA on mitochondria during this process is unknown.

The U.S. Environmental Protection Agency and the European Food Safety Authority have established tolerable daily intake (TDI), or reference dose, of 50 µg/kg/day for BPA as a ‘safe dose’. However, many researchers have suggested that BPA at such dose cause a variety of adverse effects [26]. Fetuses or newborns are more sensitive than adults, and exposure to chemicals during critical stages of development may cause irreversible long-lasting consequences [27]. In this study, we investigated the effect of perinatal exposure to BPA at TDI on the apoptotic index in the liver of rat offspring. In addition, the direct effect of BPA on the hepatic mitochondria was studied by using an *in vitro* apoptotic system.

## Materials and Methods

### *In vivo* Studies

#### Animals and treatments

All rats used in this study were treated humanely and with regard for alleviation of suffering, and the experimental protocols were approved by the Ethics Committee of Tongji Medical College (Permit Number: 2011-s2456). Virgin female (270–300 g) and male (350–400 g) genitor Wistar rats were purchased from Hubei Research Center of Laboratory Animal (Wuhan, China) and then housed in special pathogen-free conditions of 25±2°C temperature, 60–70% humidity and a controlled 12-h light/dark cycle. All animals were fed standard rodent chow [10] (ABSL-3 Lab of Wuhan University, Wuhan, China) and water *ad libitum*. Polypropylene plastic cages and glass water bottles were used in this study.

The day sperm-positive smear was determined as gestational day (GD) 0. Pregnant females were housed individually and randomly assigned to two groups that received either corn oil (Sigma-Aldrich, St. Louis, MO, USA) as the control or 50 µg/kg/day of BPA (Sigma-Aldrich, St. Louis, MO, USA) dissolved in corn oil by oral gravage from GD 0 to the end of lactation at postnatal day 21. The pups were given normal drinking water and fed standard rodent chow after weaning. At 3, 15 and 21 weeks, perinatally BPA-treated and control pups (n = 6 male rats in each group, belonging to different litters) were randomly selected, weighted and killed by decapitation.

#### Determination of BPA concentration in liver

Liver samples were removed from pups of the BPA-treated group and the control at birth day for analysis of BPA internal exposure levels. Details of sample pretreatments of the solid-phase extracts and the analysis of total BPA concentration by liquid chromatography–tandem mass spectrometry (LC-MS/MS) were in accordance with a previous study [28].

#### Liver sample collection and serum assays

Liver samples were removed from the pups and weighed, and then the samples were snap frozen at approximately −80°C until processing for analysis. Liver triglyceride (TG) was measured using a TG assay kit (Applygen Technologies Co. Ltd, Beijing, China), and the values were normalized to protein concentrations using the BCA protein quantitative assay kit (Thermo-Fisher, Waltham, MA, USA). Serum alanine aminotransferase (ALT) was measured using standard biochemical kits (BioVision, Milpitas, USA) according to the manufacturer’s instructions.

#### Histological analysis

Terminal deoxynucleotide transferase-mediated deoxy-UTP nick end labeling (TUNEL) assay was used to identify double-stranded DNA fragmentation and the characteristics of DNA degradation by apoptosis. For the TUNEL staining, the standard protocol for paraffin sections was performed according to the manufacturers’ instructions (Roche Applied Science, IN, USA). Apoptotic cells were counted in a selected microscopic field per liver section by an experienced pathologist blind to the experimental protocol. The apoptosis index was expressed as a percentage of TUNEL-positive cells in 1000 cells counted in the same section [29]. Nuclear condensation and fragmentation typical of apoptotic cells were further assessed by using 33258 staining kit (Beyotime Institute of Biotechnology, Shanghai, China). DNA staining was viewed by fluorescence microscopy. In addition, liver sections were stained with Oil-Red-O to visualize lipid accumulation.

#### Measurement of caspase-3 and -9 activities

Activities of casase-3 and -9 of liver cells were determined using the caspase-3 and caspase-9 activity kits (Beyotime, Beijing, China) according to the manufacturer’s instructions.

#### Immunoblotting for Bax, Bcl-2 and the release of Cytc

Total cytosolic proteins for analysis of the levels of Bax and Bcl-2, and the cytosolic proteins without mitochondrial fraction for analysis of the release of Cytc were prepared as previously described [30]. Protein samples (40 µg per lane) were subjected to 10% sodium dodecyl sulfate-Polyacrylamide gel electrophoresis and transferred onto polyvinylidene fluoride membranes (Bio-Rad, Hercules, CA, USA). Membranes were incubated with specific primary antibodies Bax, Bcl-2, Cytc (1∶1000 diluted, Cell Signaling Technology, MA, USA) followed by incubation with horseradish peroxidase-conjugated secondary antibody. The intensity range of immunostaining was detected using the ECL chemiluminescence system (Thermo Fisher Scientific inc., Waltham, MA, USA).

### *In vitro* Studies

#### Isolation of mitochondria and BPA treatment

To study the direct effect of BPA on mitochondria, liver mitochondria were isolated from untreated neonatal rats as described previously [30]. The mitochondria were purified by differential centrifugation and separation on a sucrose gradient (1.0 M/1.5 M). Then, the mitochondria were resuspended in a buffer (5 mg/mL bovine serum albumin, KH_2_PO_4_, 400 mM Mannitol, and 50 mM Tris HCl, pH 7.4) and kept on ice for up to 4 hr. The mitochondria were treated with 3% ethanol as the control, 1.47 ng/mL BPA or 2.60 ng/mL BPA β-d-glucuronide (Santa Cruz Biotechnology, CA, USA) dissolved in 3% ethanol.

#### Electron microscopy of isolated mitochondria and mitochondrial swelling assay

For mitochondrial swelling assay, mitochondria were washed and then resuspended with 0.025 M Tris HCl containing 0.175 M KCl (pH 7.4). Mitochondrial swelling, an indicator of mitochondrial permeability transition (PT), was measured by the decrease in the absorbance at 520 nm using a spectrophotometer after different time intervals in a temperature-controlled cell. To examine whether a change in MPT results in structural alterations, electron microscopic assessment was undertaken. The mitochondrial pellet was fixed in PBS-buffered 1% gluataraldehyde/1% paraformaldehyde, and examined by transmission electron microscopy (Tecnai G2 12, FEI, Netherland).

#### Isolation of nuclei and the morphologic features determination

Rat liver nuclei were isolated using a previously described standard method [31]. After mitochondria isolated from neonatal rat liver were treated with BPA at 30°C for 55 mins, reaction mixtures were centrifuged and then supernatants containing proteins released from mitochondria were collected as described previously [30]. For nuclear morphologic features assessment, 6 µl of liver nuclei, 1 mM MgCl_2_, 1 mM dATP (deoxyadenosine 5′-triphosphate) and 50 µl of HeLa cytosol (250 g) were incubated with the BPA-treated mitochondrial supernatant at 37°C for 2 hr. Cyt c (Sigma, Santa Clara, CA, USA) was used as the positive control, and the supernatant from untreated mitochondira was the negative control. The direct effect of BPA (1.47 ng/mL) on the isolated liver nuclei was also examined. After nuclei were stained with propidium iodide (PI), nuclear morphologic features were examined with a fluorescence microscope (Invitrogen, F14942, Carlsbad, CA, US).

### Statistical Analysis

Data are expressed as means ± SEM and analyzed using SPSS 13.0 (SPSS, Chicago, IL). The changes over time were analyzed by repeated measures analysis of variance (ANOVA), and comparisons between the control and BPA-treated group at each time studied were analyzed using multivariate analysis of variance (MANOVA) followed by Bonferroni t-tests. The other data were analyzed with ANOVA. A *P*-value of <0.05 was considered statically significant.

## Results

### *In vivo* Studies

#### Perinatal exposure to BPA increases liver apoptosis in rat offspring

After perinatal exposure to BPA at the TDI, body weight gain of the pregnant rats, litter size and sex ratio showed no significant change compared to controls. The mean concentration of total BPA in the liver of the BPA-treated neonatal rats was 1.47±0.39 ng/g, while BPA was detected under the detection limit in the control rats.

**Figure 1:**
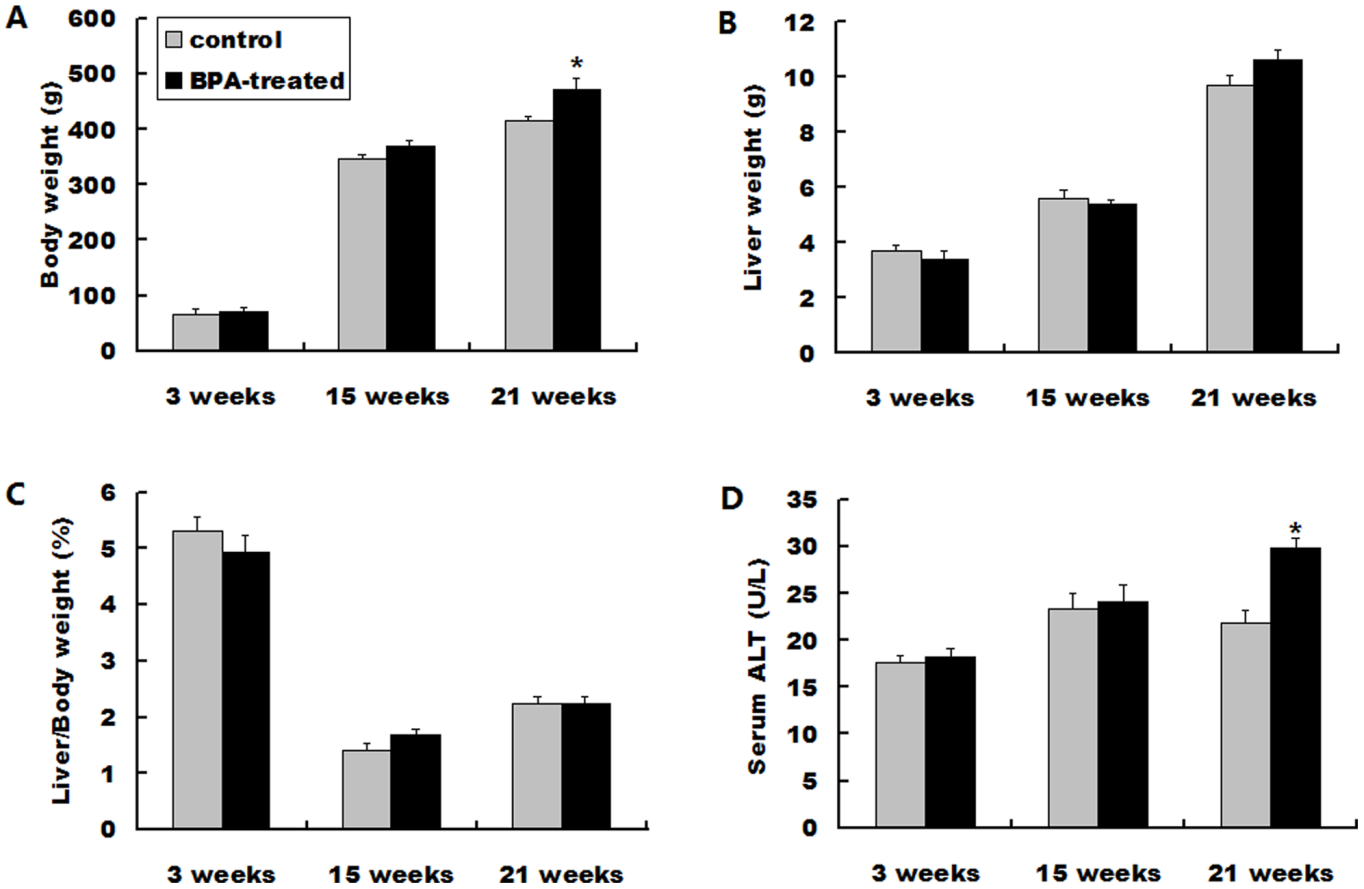
Body weight, liver weight, liver/body weight ratio and serum ALT of the rat offspring at 3, 15 and 21 weeks. (A) Body weight. (B) Liver weight. (C) Live/body weight ratio. (D) Serum ALT levels. Data represent as means ± S.E.M. (n = 6 rats per group, only 1 offspring was selected per litter). *P<0.05 denotes significant difference compared with controls.

Body weight in the BPA-treated offspring and the control were increased in time trend and perinatal exposure to BPA significantly increased body weight at 21 weeks compared with the control group (*P*<0.05) (Fig. 1A). The liver weight were also increased over time, and there’re no significant changes between the two groups at each age studied (Fig. 1B). The relative liver/body weight of the BPA-treated and control offspring remained similar throughout all ages studied (Fig. 1C). Liver injury was assessed by determining serum ALT, and time-course of BPA-increased serum ALT levels was observed. Perinatal exposure to BPA only resulted in a minor increase in serum ALT levels at 3 and 15 weeks but a significant increase at 21 weeks compared to the control (*P*<0.05) (Fig. 1D).

**Figure 2:**
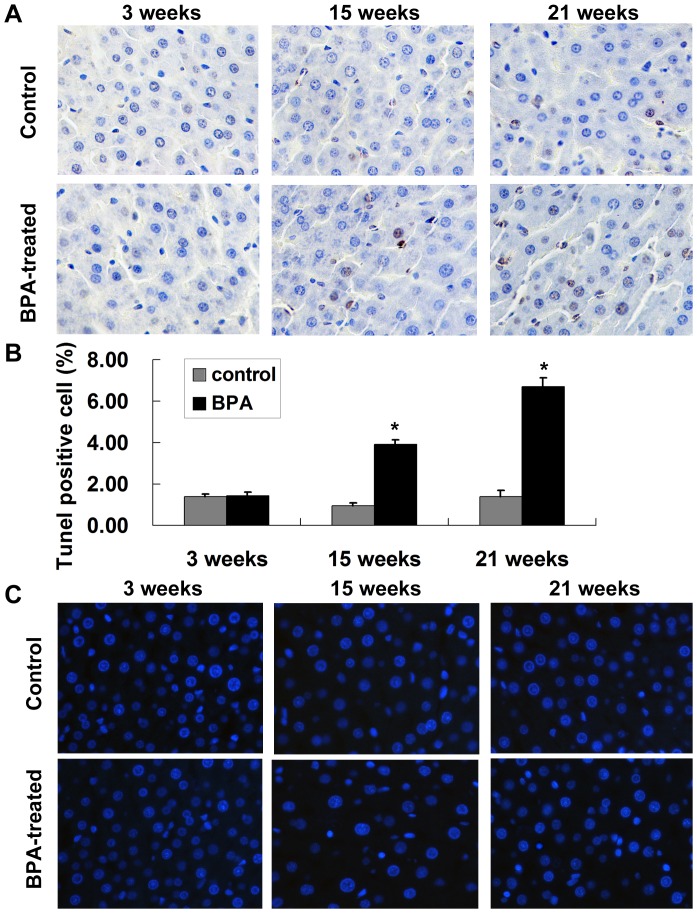
Perinatal exposure to BPA increases hepatocyte apoptosis of rat offspring. (A) Representative images of the TUNEL assay on liver tissue of the control and BPA-treated offspring at 3, 15 and 21 weeks. The TUNEL positive cells increased in the liver of BPA-treated offspring at 21 weeks (magnification, 400×). (B) Percentages of apoptotic cells in the livers from the control group and BPA-treated offspring. Percentages of apoptotic cells were quantified by counting TUNEL-positive cells in 1000 cells in a selected microscopic field per liver section. Data are means ± S.E.M. (n = 6 rats per group; only 1 offspring was selected per litter). *P<0.05 compared with controls. (C) Representative images of Hoechst 33258-stained sections of liver tissue from rats of respective group (magnification, 400×).

Hepatocyte apoptosis in liver tissue samples was examined using TUNEL assay. Perinatal exposure to BPA increased liver TUNEL-positive cells in the offspring with time, while a significant increase was observed only at 15 and 21 weeks compared with controls (*P*<0.05) (Fig. 2A, B). Hoechst stained liver sections also revealed more apoptosis and chromatin condensation in the BPA-treated rats compared with the control at 15 and 21 weeks (Fig. 2C). Moreover, the BPA-treated rats exhibited a greater accumulation of lipids in the liver and significantly elevated hepatic TG at 27 weeks compared with the control; however, significant hepatic lipid accumulation was not present in 21-week-old BPA-treated rats (Figure S1).

#### Perinatal exposure to BPA activates caspase-3 and -9 in liver tissue

**Figure 3:**
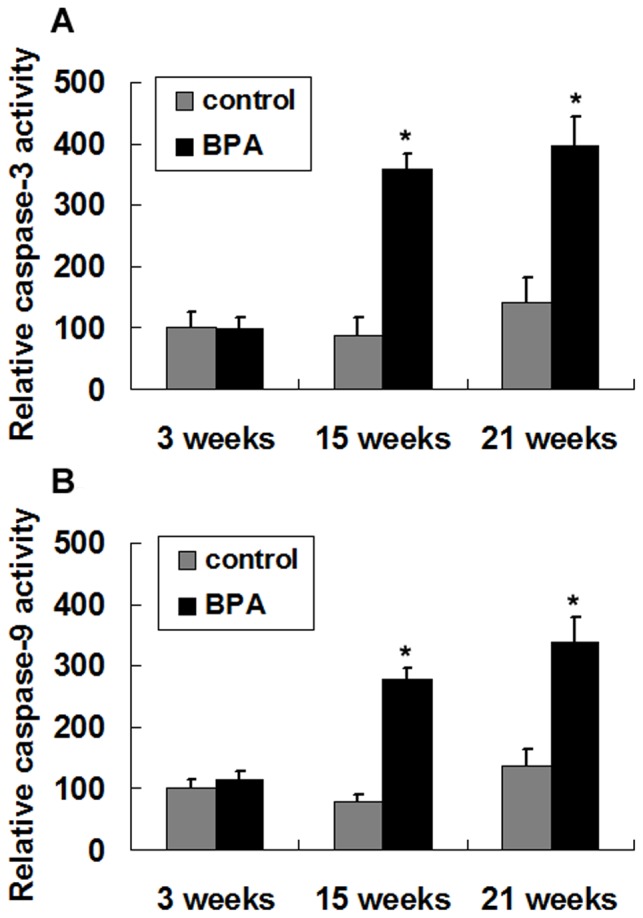
Activity of caspase-3 and caspase-9 in liver tissue after perinatal exposure to BPA. Activity of caspase-3 (A) and caspase-9 (B) in the liver of 3-week, 15-week and 21-week rat offspring were measured. Values are means ± S.E.M. (n = 6 rats per group; only 1 offspring was selected per litter). *P<0.05 compared with controls.

As capase-3 is the main effector caspase that is involved in apoptosis, we examined caspase-3 activity in the rat liver. Consistent with the TUNEL results, the BPA-treated offspring showed significantly higher activity of caspase-3 than the corresponding control group at 15 and 21 weeks (*P*<0.05) (Fig. 3A).

Due to the alteration in mitochondrial function caused by BPA was reported previously [19], [21], the elevated caspase-3 activity observed in this study suggests that the initiator caspase-9 is activated via the mitochondrial pathway. Accordingly, we tested caspase-9 activity and found significant higher levels of caspase-9 activity in BPA-treated group at 15 and 21 weeks compared to the corresponding control group at each age (*P*<0.05) (Fig. 3B).

#### Perinatal exposure to BPA alters protein levels of Cyt c and the Bcl-2 family in rat liver

**Figure 4:**
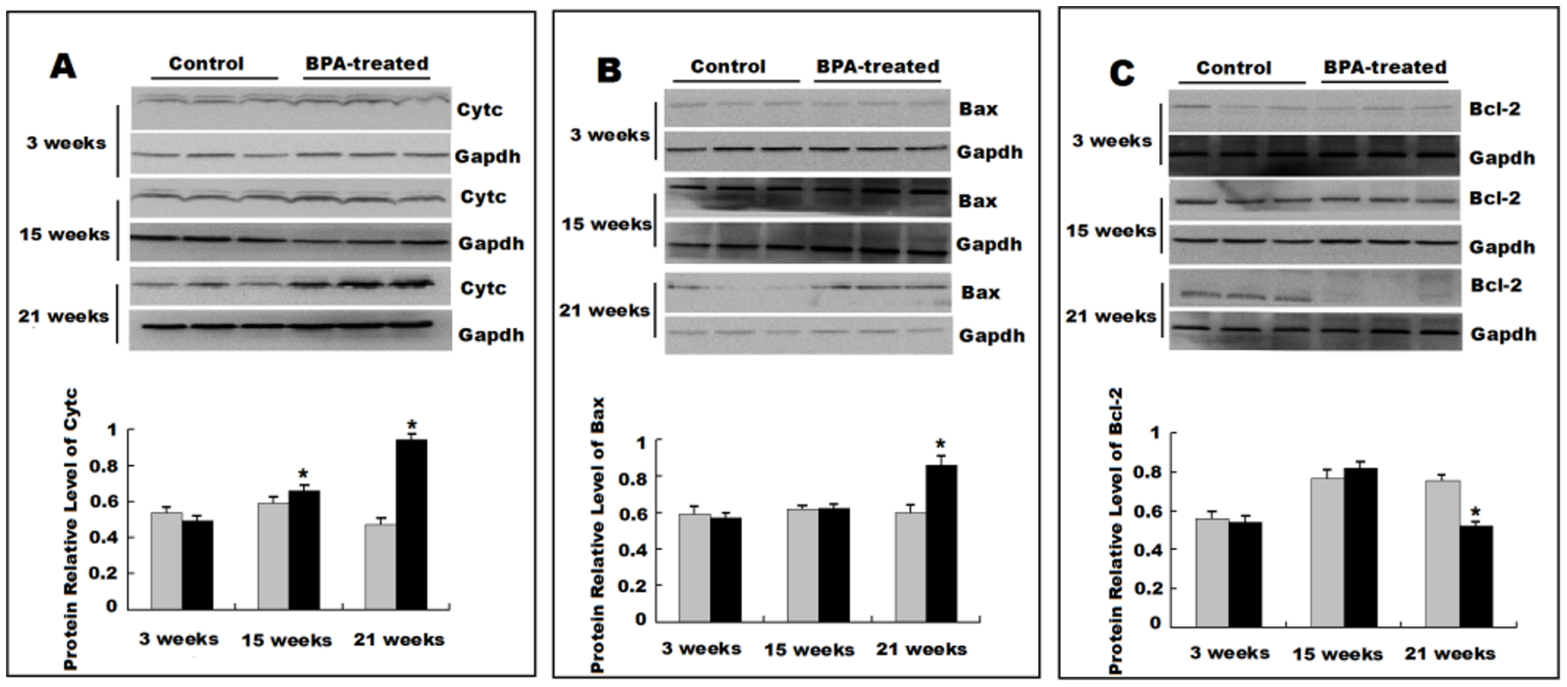
Perinatal exposure to BPA alters the levels of Cyt c, Bcl-2 and Bax. (A) Western blot analysis of the Cyt c release from mitochondria into the cytosol in rat livers at 3 and 21 weeks. Western blotting analysis of Bax (B) and Bcl-2 (C) in rat offspring liver at 3, 15 and 21 weeks. The protein expression levels were estimated by densitometry with Gapdh as an internal control. Data are expressed as means ± S.E.M. for three independent experiments (n = 6 rats per group; only 1 offspring was selected per litter).). *P<0.05 compared with controls.

Activation of caspase-9 in liver cells of BPA-treated rat offspring suggests an involvement of mitochondria-dependent apoptotic pathway. Leakage of Cyt c from mitochondria is considered as a major event in cell apoptosis and may be accompanied by expression changes of pro- and anti-apoptotic members of the Bcl-2 family [24]. We evaluated the leakage of Cyt c from the mitochondria and the protein levels of Bax and Bcl-2 in the liver by Western blotting. Significantly increased leakage of Cyt c from the mitochondria was observed in liver from 15-weeks and 21-weeks rats with perinatal exposure to BPA compared to the corresponding control group at each age (*P*<0.05) (Fig. 4A). The levels of pro-apoptotic protein Bax were significantly increased in the liver of the BPA-treated rats at 21 weeks (*P*<0.05) (Fig. 4B), and the levels of anti-apoptotic protein Bcl-2 were significantly decreased in the BPA group at 21 weeks (*P*<0.05) (Fig. 4C).

### *In vitro* Studies

Activation of caspases and the leakage of Cyc from mitochondria strongly implicate an impact of BPA on mitochondria to initiate hepatocyte apoptosis. To explore whether there was a cause-and-effect relationship, an *in vitro* study was performed. Liver mitochondria were isolated from untreated neonatal rats and then exposed to BPA at the dose which is equivalent to the internal exposure level of BPA detected in animal model.

#### BPA induces changes in mitochondrial PT and morphologic features

**Figure 5:**
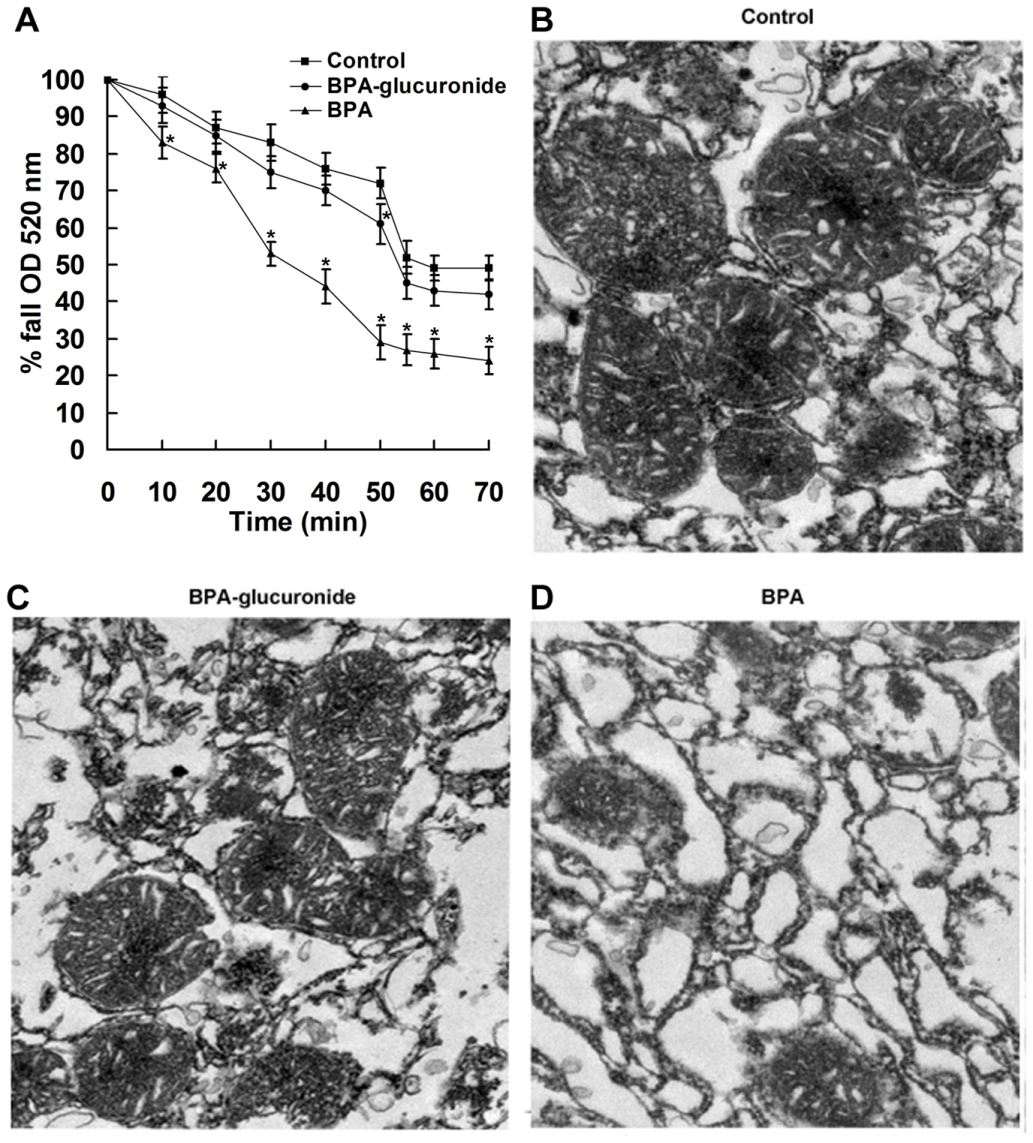
Effect of BPA on mitochondrial permeability transition (PT) and morphologic features of isolated liver mitochondria. (A) Changes of PT in the isolated mitochondria with time. Mitochondria isolated from neonatal rat liver were treated with BPA (0 and 1.47 ng/mL) or BPA-glucuronide (2.60 ng/mL). The decrease in optical density measured at 520 nm with time was normalized to 100. Data are shown of three independent experiments. Ultrastructural features of mitochondria treated with the control (B), BPA-glucuronide (C) and BPA (D). Representative images of transmission electron microscopy were shown (magnification, 10,000×).

As the PT is associated with swelling of the mitochondrial matrix, to determine whether BPA induces PT, we determined the changes in volume of the isolated liver mitochondria by measuring the change of optical absorbance. BPA treatment (1.47 ng/mL) led to slow swelling of isolated liver mitochondria with time and induced a significant decrease in optical density from 20 min compared with the control (*P*<0.05) (Fig. 5A). After incubation of BPA for 55 min, swelling was sustained there-after. In addition, to examine whether BPA-glucuronide, the majority of conjugated BPA form, also has the similar effect on mitochondria PT, we used the same molar concentration of BPA-glucuronide (2.60 ng/mL) to test. BPA-glucuronide only induced a mild decrease in the optical density compared to the control (Fig. 5A).

From electron microscopic assessment, the control mitochondria showed regular cristae and integrated morphologic features (Fig. 5B), most of the mitochondria treated BPA-glucuronide were also normal (Fig. 5C). However, mitochondria treated with BPA were swollen, and outer membranes were enlarged and ruptured (Fig. 5D). BPA significantly increased mitochondria with abnormal morphologic features (50±4.6%) compared with the control (15±2.9%) (*P*<0.05), while BPA-glucuronide only induced 19±3.2% abnormal mitochondria.

#### BPA-induced release of proteins from the mitochondria initiates apoptosis in nuclei

**Figure 6:**
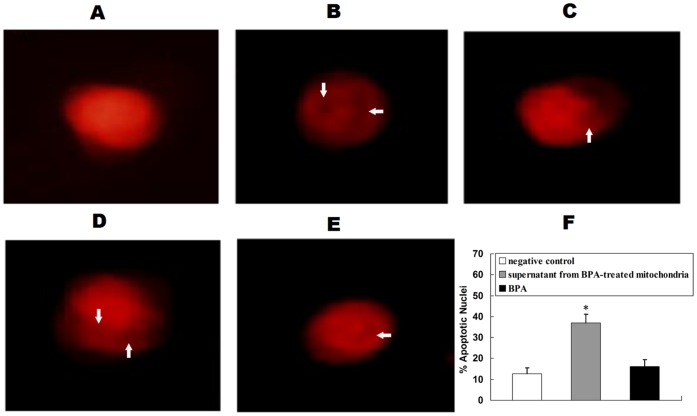
BPA-induced release of proteins and the initiation of apoptosis *in vitro*. After liver nuclei were incubated with the supernatant from the mitochondria treated with BPA, negative control, positive control or BPA, nuclear morphologic features were assessed. (A) Normal morphologic features; (B) Positive control; (C–E) Various stages of apoptosis in liver nuclei after incubation with BPA-treated mitochondrial supernatant, showing condensation or fragmentation of chromatin in nuclei (arrow); Representative images of three separate experiments are shown (magnification, 400×). (F) Changes of apoptotic nuclei. The percentage of apoptotic nuclei was counted in five different fields of view. The results are expressed as means±S.E.M. The experiment was repeated three times. *P<0.05 compared with the negative control.

The induction of PT induced by BPA suggests that possible apoptogenic proteins are released from the damaged mitochondria. In order to investigate if those proteins could initiates apoptosis in nuclei, the liver nuclei were isolated and incubated with proteins released from the mitochondria that had been treated with BPA, and apoptotic morphologic features stained with PI were assessed by a florescence microscope. The effect of BPA alone on nuclei was also investigated. The negative control showed normal morphologic features of liver nuclei with evenly distributed chromatin (Fig. 6A) and the positive control showed irregular condensations of chromatin in nuclei (Fig. 6B). After the liver nuclei were incubated with BPA-treated mitochondrial supernatant, condensed or fragmented nuclei characteristic of apoptosis were observed (Fig. 6C–E). The supernatant from BPA-treated mitochondria significantly increased the percentage of apoptotic nuclei (37.0%±4.2%) compared to the negative control (12.8%±2.6%) (Fig. 6F), while BPA only induced a mild increase in apoptotic nuclei (16.4%±3.3%).

## Discussion

Many studies have investigated the long-lasting effects of BPA exposure at early development stages on the reproductive tract [7], [8], [32], [33], neural behaviors [34], [35] and metabolic function [9], [10], [12]. However, little data on the long-term effects of early BPA exposure at environmentally relevant dose on liver tissue were available. Although the dose of BPA we chosen in this study (50 µg/kg/day) is higher than human daily intake, the average total BPA concentration in the liver of BPA-treated rats (1.47 ng/g) was in the range of that reported in human fetal livers [5]. This is may be due to the fact that BPA metabolism in rodents differs from that in humans [36]. Thus, the dose we used in this study can be considered environmentally relevant. Here, we provide experimental evidence that early BPA exposure at environmentally relevant dose induces long-term injury on liver, and the activation of mitochondrial pathway of hepatocyte apoptosis may be involved in this process.

In the present study, we observed that perinatal exposure to BPA significantly increased the body weight of pups at adulthood, and the results are in accordance with some previous studies demonstrating increased postnatal growth in rodents prenatally or perinatally exposed to BPA at low doses [11], [37]. Although the liver weight and liver/body weight ratio of BPA-treated pups didn’t differ from controls, the significant increase in serum ALT levels at 21 weeks indicated that liver injury induced by BPA. Then, we assessed liver cell apoptosis and found an extremely significant increase in TUNEL positive cells in BPA-treated rats at 15 and 21 weeks, which accompanied by a modest increased level of serum ALT observed at 21 weeks. Serum ALT value has long been used as a marker of liver injury. However, actually, in some chronic liver injury, there is no apparent change in ALT observed. Some studies have demonstrated that the serum ALT values do not correlate with the severity of the histopathological changes and the parameters of apoptosis in liver disease, and suggest histological assessment can more accurately determine the disease severity [38].

Hepatic homeostasis is achieved through a regular cell turnover involving apoptosis of hepatocytes, and increased apoptosis of hepatocytes is considered to be an important mechanism contributing to various kinds of liver diseases [39]. The induction of apoptosis is correlated with the activation of caspases, which have proteolytic activity and are able to cleave proteins to result in cell shrinkage, chromatin condensation and DNA fragmentation [40]. We observed the significant increases in the activity of caspase-3 in BPA-treated offspring at 15 and 21 weeks compared to the corresponding control group, suggesting that apoptosis is caspase dependent. Caspase-3, a key molecular marker of apoptotic signaling, is activated by either mitochondria dependent or death receptor dependent apoptotic pathways. Previous researches reported that BPA induced mitochondria dysfunction in the isolated rat hepatocytes [19] and human HepG2 cell [21], which led us to study the role of mitochondria played in liver cell apoptosis induced by BPA. Consistent with the change of caspase-3 activity, the further findings of elevated levels of caspase-9 activity and cytosolic Cyt c in hepatocytes of BPA-treated pups at 15 and 21 weeks indicated an involvement of a mitochondrial pathway of apoptosis.

The Bcl-2 family of proteins, containing both pro-apoptotic and anti-apoptotic members, are known to regulate mitochondrial-mediated apoptosis [41]. However, we only found significant change in the expression of antiapoptotic Bcl-2 proteins and the proapoptotic Bax proteins in BPA-treated group at 21 weeks. It has been reported that some drug or hormone can enforce or inhibit mitochondrial PT and then induce or prevent apoptosis [30], [42]. The induction of the PT, an important indicator of mitochondrial integrity and function, can result in mitochondrial swelling and the release of apoptogenic proteins from mitochondria [43]. The so-called PT is due to the opening of a regulated proteaceous pore, also called PT pore. The PT pore complex, which has been proposed located with inner/outer membrane contact sites, are viewed as an integrator that senses metabolic perturbations [42]. Perinatal exposure to BPA may act on the liver mitochondria in a direct or an indirect fashion to provoke PT first, causing release of apoptogenic proteins and activation of caspase.

Because the mechanism of BPA impacts on liver cells *in vivo* is complicated, an *in vitro* system was used to study the direct effect of BPA on liver mitochondria to minimize the systemic effects. We found that BPA induced a significant increase in mitochondrial PT coupled with ultrastructural changes in morphologic mitochondrial features, indicating a direct effect of BPA on mitochondria. In addition, the release of apoptogenic proteins from the mitochondria induced by BPA was further demonstrated to cause condensed chromatin in isolated liver nuclei.

We also investigated whether BPA-glucuronide has the similar effect of BPA on the isolated mitochondria, since BPA is metabolized into its glucurono-conjugated form *in vivo* [13] and glucuronidation is considered to be a detoxification mechanism [44]. Our results showed that the toxic effects of BPA are greater than those of its glucuronide metabolite. Thus, determination of both free and conjugated BPA may be more appropriate for risk assessment, but there is limited data currently since the quantification method continues to present challenges [45].

It’s worth noting that the increased apoptosis induced by BPA took several weeks to become visible *in vivo*, but the *in vitro* results demonstrated that BPA had a direct effect on mitochondria. There are two reasons may explain the discrepancy. *In vivo*, the apoptotic cells will soon be phagocytized by the neighboring macrophage [46]. When the injury induced by low dose of BPA was not serious, we could not observe a significant increase in apoptosis cells. But it did not mean there was no potential injury. Some studies reported that early environmental stress during the initial stages of development could set a precedent for “priming” of the mitochondria to cause changes in mitochondria observed in adulthood [47], [48]. Although the rat pups in our study were not treated with BPA after weaning and the metabolic clearance rate of BPA is only several hours [49], the cell apoptosis in liver and associated markers of mitochondria-dependent pathway become more serious with increasing age, demonstrating BPA has a long lasting effect through a direct or an indirect way. Thus, more early sensitive markers associated with mitochondrial injury should be explored in the future studies for better understanding the effect of BPA on mitochondrial *in vivo*.

Alterations in mitochondrial function have been implicated in the pathogenesis of hepatic steatosis which is also associated with hepatocyte apoptosis, and a recent *in vitro* study reported that BPA-induced lipid accumulation in HepG2 cells by disturbing mitochondrial function [21]. Therefore, we assessed hepatic lipid accumulation by Oil-Red-O staining and measuring hepatic TG. Significantly higher hepatic TG accumulation was observed in BPA-treated rats at the age of 27 weeks compared to the control group. The novel finding suggests that mitochondrial injury induced by perinatal exposure to BPA may also contribute to the later onset of hepatic steatosis, which needs further investigation.

In conclusion, this study demonstrates that BPA induces mitochondria-mediated apoptosis in hepatic cells, providing an explanation for long-term liver injury associated with early-life BPA exposure. In addition, our *in vitro* data indicate that BPA acts directly on mitochondria, altering mitochondrial ultrastructure, inducing PT and releasing proteins that lead to activation of apoptosis.

## Supporting Information

Figure S1 **Effect of perinatal exposure to BPA on lipid accumulation in the livers.** (A) Representative images of Oil-Red-O staining of neutral lipids presented on sections of livers from the control and BPA-treated rats at 21 and 27 weeks. Neutral lipids appear in red (magnification, ×400). (B) Quantified analysis of TG in liver. Data are means ± S.E.M. (n = 6 rats per group; only 1 offspring was selected per litter). *P<0.05 compared with controls.
(TIF)

## References

[1] Schonfelder G, Wittfoht W, Hopp H, Talsness CE, Paul M, et al (2002) Parent bisphenol A accumulation in the human maternal-fetal-placental unit. Environ Health Perspect 110: A703–707. 12417499 10.1289/ehp.110-1241091 PMC1241091

[2] Wan Y, Choi K, Kim S, Ji K, Chang H, et al (2010) Hydroxylated polybrominated diphenyl ethers and bisphenol A in pregnant women and their matching fetuses: placental transfer and potential risks. Environ Sci Technol 44: 5233–5239. 20509646 10.1021/es1002764

[3] Ikezuki Y, Tsutsumi O, Takai Y, Kamei Y, Taketani Y (2002) Determination of bisphenol A concentrations in human biological fluids reveals significant early prenatal exposure. Hum Reprod 17: 2839–2841. 12407035 10.1093/humrep/17.11.2839

[4] Yamada H, Furuta I, Kato EH, Kataoka S, Usuki Y, et al (2002) Maternal serum and amniotic fluid bisphenol A concentrations in the early second trimester. Reprod Toxicol 16: 735–739. 12401500 10.1016/s0890-6238(02)00051-5

[5] Cao XL, Zhang J, Goodyer CG, Hayward S, Cooke GM, et al (2012) Bisphenol A in human placental and fetal liver tissues collected from Greater Montreal area (Quebec) during 1998–2008. Chemosphere 89: 505–511. 22682542 10.1016/j.chemosphere.2012.05.003

[6] Sun Y, Irie M, Kishikawa N, Wada M, Kuroda N, et al (2004) Determination of bisphenol A in human breast milk by HPLC with column-switching and fluorescence detection. Biomed Chromatogr 18: 501–507. 15386523 10.1002/bmc.345

[7] Prins GS, Birch L, Tang WY, Ho SM (2007) Developmental estrogen exposures predispose to prostate carcinogenesis with aging. Reprod Toxicol 23: 374–382. 17123779 10.1016/j.reprotox.2006.10.001 PMC1927084

[8] Suzuki A, Sugihara A, Uchida K, Sato T, Ohta Y, et al (2002) Developmental effects of perinatal exposure to bisphenol-A and diethylstilbestrol on reproductive organs in female mice. Reprod Toxicol 16: 107–116. 11955941 10.1016/s0890-6238(02)00005-9

[9] Alonso-Magdalena P, Vieira E, Soriano S, Menes L, Burks D, et al (2010) Bisphenol A exposure during pregnancy disrupts glucose homeostasis in mothers and adult male offspring. Environ Health Perspect 118: 1243–1250. 20488778 10.1289/ehp.1001993 PMC2944084

[10] Wei J, Lin Y, Li Y, Ying C, Chen J, et al (2011) Perinatal exposure to bisphenol A at reference dose predisposes offspring to metabolic syndrome in adult rats on a high-fat diet. Endocrinology 152: 3049–3061. 21586551 10.1210/en.2011-0045

[11] Somm E, Schwitzgebel VM, Toulotte A, Cederroth CR, Combescure C, et al (2009) Perinatal exposure to bisphenol a alters early adipogenesis in the rat. Environ Health Perspect 117: 1549–1555. 20019905 10.1289/ehp.11342 PMC2790509

[12] Liu J, Yu P, Qian W, Li Y, Zhao J, et al (2013) Perinatal bisphenol A exposure and adult glucose homeostasis: identifying critical windows of exposure. PLoS One 8: e64143. 23675523 10.1371/journal.pone.0064143 PMC3651242

[13] Pottenger LH, Domoradzki JY, Markham DA, Hansen SC, Cagen SZ, et al (2000) The relative bioavailability and metabolism of bisphenol A in rats is dependent upon the route of administration. Toxicol Sci 54: 3–18. 10746927 10.1093/toxsci/54.1.3

[14] Lang IA, Galloway TS, Scarlett A, Henley WE, Depledge M, et al (2008) Association of urinary bisphenol A concentration with medical disorders and laboratory abnormalities in adults. Jama 300: 1303–1310. 18799442 10.1001/jama.300.11.1303

[15] Bindhumol V, Chitra KC, Mathur PP (2003) Bisphenol A induces reactive oxygen species generation in the liver of male rats. Toxicology 188: 117–124. 12767684 10.1016/s0300-483x(03)00056-8

[16] Hanioka N, Jinno H, Nishimura T, Ando M (1998) Suppression of male-specific cytochrome P450 isoforms by bisphenol A in rat liver. Arch Toxicol 72: 387–394. 9708877 10.1007/s002040050518

[17] Marmugi A, Ducheix S, Lasserre F, Polizzi A, Paris A, et al (2012) Low doses of bisphenol A induce gene expression related to lipid synthesis and trigger triglyceride accumulation in adult mouse liver. Hepatology 55: 395–407. 21932408 10.1002/hep.24685

[18] Ronn M, Kullberg J, Karlsson H, Berglund J, Malmberg F, et al (2013) Bisphenol A exposure increases liver fat in juvenile fructose-fed Fischer 344 rats. Toxicology 303: 125–132. 23142792 10.1016/j.tox.2012.09.013

[19] Nakagawa Y, Tayama S (2000) Metabolism and cytotoxicity of bisphenol A and other bisphenols in isolated rat hepatocytes. Arch Toxicol 74: 99–105. 10839477 10.1007/s002040050659

[20] Ooe H, Taira T, Iguchi-Ariga SM, Ariga H (2005) Induction of reactive oxygen species by bisphenol A and abrogation of bisphenol A-induced cell injury by DJ-1. Toxicol Sci 88: 114–126. 16093527 10.1093/toxsci/kfi278

[21] Huc L, Lemarie A, Gueraud F, Helies-Toussaint C (2012) Low concentrations of bisphenol A induce lipid accumulation mediated by the production of reactive oxygen species in the mitochondria of HepG2 cells. Toxicol In Vitro 26: 709–717. 22515966 10.1016/j.tiv.2012.03.017

[22] Vaux DL (2011) Apoptogenic factors released from mitochondria. Biochim Biophys Acta 1813: 546–550. 20713095 10.1016/j.bbamcr.2010.08.002

[23] Kroemer G, Galluzzi L, Brenner C (2007) Mitochondrial membrane permeabilization in cell death. Physiol Rev 87: 99–163. 17237344 10.1152/physrev.00013.2006

[24] Kluck RM, Bossy-Wetzel E, Green DR, Newmeyer DD (1997) The release of cytochrome c from mitochondria: a primary site for Bcl-2 regulation of apoptosis. Science 275: 1132–1136. 9027315 10.1126/science.275.5303.1132

[25] Asahi J, Kamo H, Baba R, Doi Y, Yamashita A, et al (2010) Bisphenol A induces endoplasmic reticulum stress-associated apoptosis in mouse non-parenchymal hepatocytes. Life Sci 87: 431–438. 20807545 10.1016/j.lfs.2010.08.007

[26] vom Saal FS, Hughes C (2005) An extensive new literature concerning low-dose effects of bisphenol A shows the need for a new risk assessment. Environ Health Perspect 113: 926–933. 16079060 10.1289/ehp.7713 PMC1280330

[27] Newbold RR (2004) Lessons learned from perinatal exposure to diethylstilbestrol. Toxicol Appl Pharmacol 199: 142–150. 15313586 10.1016/j.taap.2003.11.033

[28] Shao B, Zhao R, Meng J, Xue Y, Wu GH, et al (2005) Simultaneous determination of residual hormonal chemicals in meat, kidney, liver tissues and milk by liquid chromatography-tamdem mass spectrometry. Analytica Chimica Acta 584: 41–50.

[29] Chen T, Zhang L, Yue JQ, Lv ZQ, Xia W, et al (2012) Prenatal PFOS exposure induces oxidative stress and apoptosis in the lung of rat off-spring. Reprod Toxicol 33: 538–545. 21440054 10.1016/j.reprotox.2011.03.003

[30] Upadhyay G, Singh R, Kumar A, Kumar S, Kapoor A, et al (2004) Severe hyperthyroidism induces mitochondria-mediated apoptosis in rat liver. Hepatology 39: 1120–1130. 15057916 10.1002/hep.20085

[31] Blobel G, Potter VR (1966) Nuclei from rat liver: isolation method that combines purity with high yield. Science 154: 1662–1665. 5924199 10.1126/science.154.3757.1662

[32] Newbold RR, Jefferson WN, Padilla-Banks E (2007) Long-term adverse effects of neonatal exposure to bisphenol A on the murine female reproductive tract. Reprod Toxicol 24: 253–258. 17804194 10.1016/j.reprotox.2007.07.006 PMC2043380

[33] Markey CM, Wadia PR, Rubin BS, Sonnenschein C, Soto AM (2005) Long-term effects of fetal exposure to low doses of the xenoestrogen bisphenol-A in the female mouse genital tract. Biol Reprod 72: 1344–1351. 15689538 10.1095/biolreprod.104.036301

[34] Fujimoto T, Kubo K, Aou S (2006) Prenatal exposure to bisphenol A impairs sexual differentiation of exploratory behavior and increases depression-like behavior in rats. Brain Res 1068: 49–55. 16380096 10.1016/j.brainres.2005.11.028

[35] Kundakovic M, Gudsnuk K, Franks B, Madrid J, Miller RL, et al (2013) Sex-specific epigenetic disruption and behavioral changes following low-dose in utero bisphenol A exposure. Proc Natl Acad Sci U S A 110: 9956–9961. 23716699 10.1073/pnas.1214056110 PMC3683772

[36] Vandenberg LN, Maffini MV, Sonnenschein C, Rubin BS, Soto AM (2009) Bisphenol-A and the great divide: a review of controversies in the field of endocrine disruption. Endocr Rev 30: 75–95. 19074586 10.1210/er.2008-0021 PMC2647705

[37] Patel BB, Raad M, Sebag IA, Chalifour LE (2013) Lifelong exposure to bisphenol a alters cardiac structure/function, protein expression, and DNA methylation in adult mice. Toxicol Sci 133: 174–185. 23418087 10.1093/toxsci/kft026

[38] Canbakan B, Senturk H, Canbakan M, Toptas T, Tabak O, et al (2010) Is alanine aminotransferase level a surrogate biomarker of hepatic apoptosis in nonalcoholic fatty liver disease? Biomarkers in medicine 4: 205–214. 20406065 10.2217/bmm.09.88

[39] Guicciardi ME, Gores GJ (2005) Apoptosis: a mechanism of acute and chronic liver injury. Gut 54: 1024–1033. 15951554 10.1136/gut.2004.053850 PMC1774601

[40] Elmore S (2007) Apoptosis: a review of programmed cell death. Toxicologic pathology 35: 495–516. 17562483 10.1080/01926230701320337 PMC2117903

[41] Shimizu S, Narita M, Tsujimoto Y (1999) Bcl-2 family proteins regulate the release of apoptogenic cytochrome c by the mitochondrial channel VDAC. Nature 399: 483–487. 10365962 10.1038/20959

[42] Susin SA, Zamzami N, Kroemer G (1998) Mitochondria as regulators of apoptosis: doubt no more. Biochim Biophys Acta 1366: 151–165. 9714783 10.1016/s0005-2728(98)00110-8

[43] Green DR, Reed JC (1998) Mitochondria and apoptosis. Science 281: 1309–1312. 9721092 10.1126/science.281.5381.1309

[44] Snyder RW, Maness SC, Gaido KW, Welsch F, Sumner SC, et al (2000) Metabolism and disposition of bisphenol A in female rats. Toxicol Appl Pharmacol 168: 225–234. 11042095 10.1006/taap.2000.9051

[45] Kosarac I, Kubwabo C, Lalonde K, Foster W (2012) A novel method for the quantitative determination of free and conjugated bisphenol A in human maternal and umbilical cord blood serum using a two-step solid phase extraction and gas chromatography/tandem mass spectrometry. J Chromatogr B Analyt Technol Biomed Life Sci 898: 90–94. 10.1016/j.jchromb.2012.04.023 22580014

[46] Dini L, Pagliara P, Carla EC (2002) Phagocytosis of apoptotic cells by liver: a morphological study. Microsc Res Tech 57: 530–540. 12112436 10.1002/jemt.10107

[47] Cotney J, McKay SE, Shadel GS (2009) Elucidation of separate, but collaborative functions of the rRNA methyltransferase-related human mitochondrial transcription factors B1 and B2 in mitochondrial biogenesis reveals new insight into maternally inherited deafness. Hum Mol Genet 18: 2670–2682. 19417006 10.1093/hmg/ddp208 PMC2701340

[48] Dillin A, Hsu AL, Arantes-Oliveira N, Lehrer-Graiwer J, Hsin H, et al (2002) Rates of behavior and aging specified by mitochondrial function during development. Science 298: 2398–2401. 12471266 10.1126/science.1077780

[49] Volkel W, Colnot T, Csanady GA, Filser JG, Dekant W (2002) Metabolism and kinetics of bisphenol a in humans at low doses following oral administration. Chem Res Toxicol 15: 1281–1287. 12387626 10.1021/tx025548t

