# Supplementary figures and images for: Early-Life Exposure to Bisphenol A Induces Liver Injury in Rats Involvement of Mitochondria-Mediated Apoptosis
Source: PLoS One. 2014 Feb 28;9(2):e90443. doi: 10.1371/journal.pone.0090443 (PMC3938763; doi:10.1371/journal.pone.0090443)

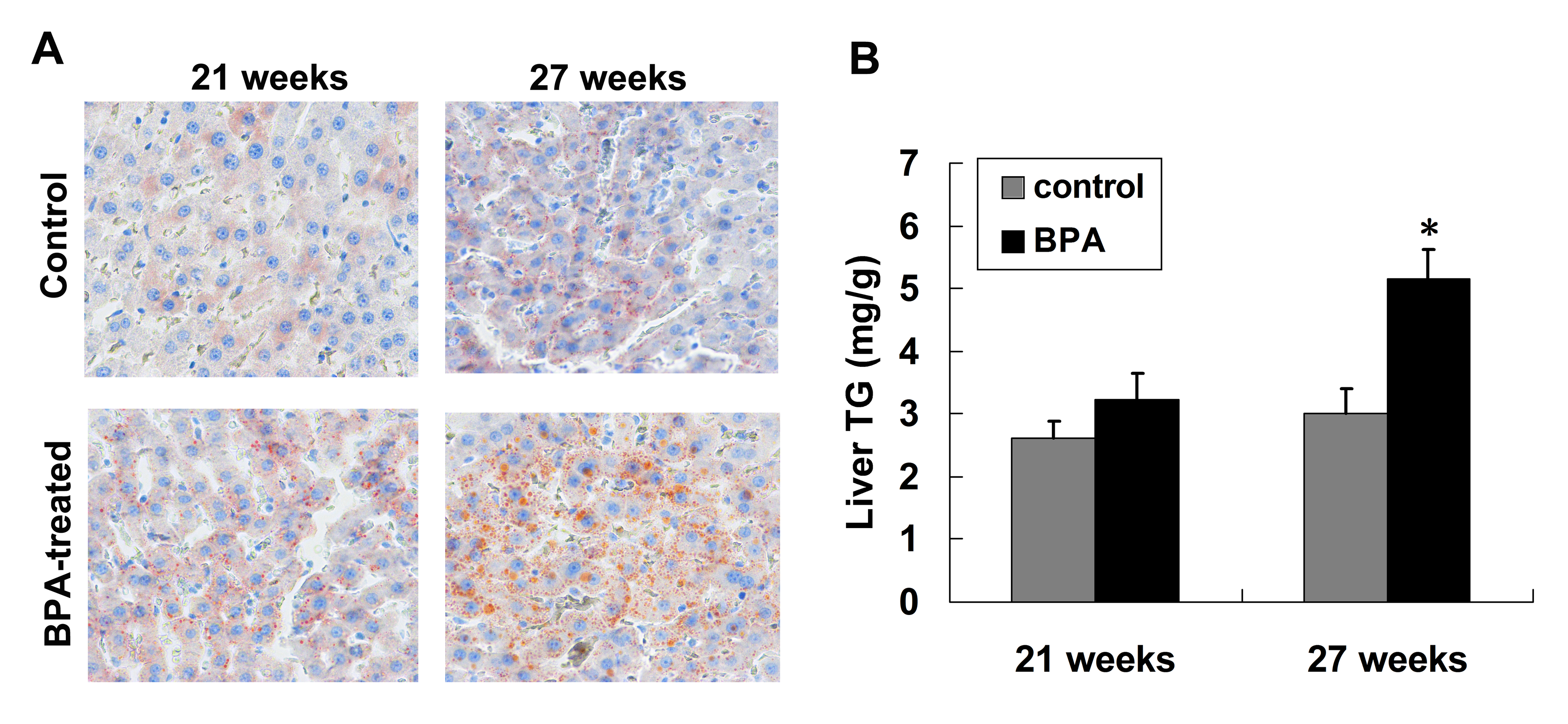

Supplement: Figure S1 — Effect of perinatal exposure to BPA on lipid accumulation in the livers. (A) Representative images of Oil-Red-O staining of neutral lipids presented on sections of livers from the control and BPA-treated rats at 21 and 27 weeks. Neutral lipids appear in red (magnification, ×400). (B) Quantified analysis of TG in liver. Data are means ± S.E.M. (n = 6 rats per group; only 1 offspring was selected per litter). *P<0.05 compared with controls. (TIF) [file pone.0090443.s001.tif]
